# Analysis of colorectal liver metastases in photon-counting detector CT - optimizing imaging through spectral reconstruction

**DOI:** 10.1186/s40644-026-01044-6

**Published:** 2026-05-07

**Authors:** Maximilian Moos, Tilman Emrich, Maximilian Nguyen, Sebastian Steinmetz, Roman Kloeckner, Felix Hahn, Paul Steiner, Constantin Scholz, Markus Moehler, Hauke Lang, Tobias Bäuerle, Tobias Jorg, Lukas Müller

**Affiliations:** 1 Department of Diagnostic and Interventional Radiology, University Medical Center of the Johannes Gutenberg University Mainz, Langenbeckstr. 1, 55131 Mainz, Germany; 2 Department of Radiology and Radiological Science, Medical University of South Carolina, Charleston, USA; 3 Department of Neuroradiology, University Medical Center of the Johannes Gutenberg University Mainz, Mainz, Germany; 4 Institute of Interventional Radiology, University Hospital Schleswig-Holstein, Lübeck, Germany; 5 Department of Internal Medicine I, University Medical Center of the Johannes Gutenberg University Mainz, Mainz, Germany; 6 Department of General, Visceral and Transplant Surgery, University Medical Center of the Johannes Gutenberg University Mainz, Mainz, Germany; 7 Research Center for Immunotherapy (FZI), University Medical Center of the Johannes Gutenberg University Mainz, Mainz, Germany

**Keywords:** Photon-counting detector CT, Colorectal liver metastases (CRLM), Virtual monoenergetic image (VMI), Iodine map, Spectral reconstruction

## Abstract

**Purpose:**

The aim of this study was to evaluate the visibility of colorectal liver metastases (CRLM) using photon-counting detector computed tomography (PCD-CT) and to determine the optimal virtual monoenergetic image (VMI) and iodine map reconstructions for improved contrast detection between metastases and surrounding liver parenchyma.

**Materials and methods:**

A total of 117 patients with 227 CRLM (up to three measurements per patient) who underwent abdominal PCD-CT for staging between 09/2022 and 08/2024 were retrospectively included. VMI were reconstructed at energy levels between 40 and 90 keV (in 10 keV increments), and scanner-generated iodine maps were additionally analysed. To quantify contrast between CRLM and liver parenchyma, the parenchyma-to-lesion ratio (PLR) was calculated for each VMI and iodine map. The contrast-to-noise ratio (CNR) was determined based on attenuation values of the metastases and the bilateral musculus erector spinae, as well as its standard deviation. For the iodine map, lesion and parenchyma iodine concentrations were used analogously. Subjective assessment of metastases visibility on the three best VMIs in PLR and CNR (40–60 keV) and iodine maps were independently performed by three radiologists.

**Results:**

Lesion and liver attenuation decreased steadily with higher keV levels. Iodine maps showed markedly higher iodine concentration in liver parenchyma than in metastases. The PLR was highest on the iodine map (3.29 ± 2.01), followed by 40 keV (2.19 ± 0.73). Regarding CNR, the 40 keV VMI showed the highest value (1.49 ± 1.70), followed by the iodine map (1.09 ± 0.99). CNR values decreased further at higher energies and significantly reduced at 70–90 keV. Paired superiority testing confirmed 40 keV as the best-performing VMI, showing significantly higher CNR than the iodine map, whereas PLR remained superior on the iodine map. Subjective ratings indicated that the 50 keV VMI provided the best visibility of CRLM. The iodine map consistently received lower subjective ratings across all criteria.

**Conclusion:**

Both iodine maps and low-keV VMIs, particularly at 40 keV, demonstrated high PLR and CNR values, contributing to improved depiction of CRLM in PCD-CT. The complementary use of these reconstructions may enhance lesion detection and overall diagnostic confidence.

## Introduction

Colorectal cancer (CRC) is one of the most prevalent malignancies worldwide and a leading cause of cancer-related morbidity and mortality [1]. In approximately 30% of CRC patients, synchronous or metachronous colorectal liver metastases (CRLM) develop, significantly affecting both overall survival and therapeutic strategies [1–3]. Early detection and accurate characterization of metastases are crucial for effective treatment planning, guiding decisions regarding resection or systemic therapies [4].

According to the ESMO guideline, contrast-enhanced computed tomography (CT) remains an important imaging modality in staging for detecting CRLM [5]. However, traditional energy-integrating detector CT (EID-CT) has notable limitations in detecting subtle CRLM, which is why MRI is recommended, particularly for patients with potentially resectable or ablation-eligible metastases, to avoid missing additional lesions [5, 6]. With the advent of photon-counting detector CT (PCD-CT), substantial improvements in spatial resolution, dose efficiency, and contrast-to-noise ratio (CNR) have been achieved compared with traditional energy-integrating detector CT (EID-CT) [7–9]. Several investigations have confirmed these benefits across various oncologic indications, for example improved detection of small lung nodules by improved spatial resolution [10–13].

Additionally, the direct detection of photons enables the acquisition and reconstruction of spectral datasets such as Virtual Monoenergetic Imaging (VMI) and quantitative iodine mapping [12, 13]. VMI, which generates images at specific energy levels, has shown potential for improving contrast differentiation between lesions and surrounding parenchyma. Lower keV settings (e.g., 40 keV) have been found to offer superior CNR of arterial phase imaging compared to EID-CT [7]. Dual-energy CT has already demonstrated that lower-keV VMIs can substantially improve the accuracy of CRLM detection [14, 15]. With PCD-CT, such VMI reconstructions are inherently available and can be generated retrospectively from every scan, thereby enhancing lesion conspicuity without requiring protocol changes at acquisition.

With PCD-CT, iodine map imaging allows for detection, differentiation, and quantification of iodine contrast agent within solid organs and lesions, with retrospective availability from routine staging examinations [16–18]. Iodine quantification is expressed as iodine concentration (IC) within the analysed organ or lesion, typically measured in mg/ml [16]. According to the literature, IC serves as a crucial quantitative parameter in assessing parenchyma perfusion and tumour characteristics [19].

However, despite promising data in hepatic lesions and phantom studies, the specific value of iodine quantification and spectral imaging for the depiction of CRLM remains underexplored. Evidence on whether PCD-CT reconstructions, such as VMI at ultra-low keV and iodine mapping, can enhance detection or lesion characterization in CRLM is still limited [20, 21]. This knowledge gap provides the rationale for our study, which aims to evaluate whether PCD-CT with optimized VMI and iodine quantification can improve lesion conspicuity, contrast-to-noise ratio, and quantitative differentiation between metastases and surrounding liver parenchyma.

## Methods

This retrospective study was approved by the local ethics committee of Rhineland-Palatinate. Informed consent was waived by the ethics committee (Reg. No. 2022–16359).

### Patients

From 09/2022 to 08/2024, a total of 117 patients with radiological confirmed CRLM who underwent PCD-CT were identified through our local radiology information system. Lesion selection was performed on a per-patient basis. In patients with multiple metastases, up to the three largest lesions were included, whereas in patients with fewer lesions, all available metastases were analyzed. Metastases that had undergone prior local therapy were excluded, resulting in a total of 227 lesions. The presence and nature of metastases were confirmed based on clinical history, histopathology, follow-up imaging including MRI or ultrasound, and multidisciplinary tumour board consensus.

Patients with metastases from other primary malignancies were excluded to ensure a homogeneous cohort of colorectal liver metastases. In addition, metastases that had undergone prior local therapy (e.g., thermal ablation) were excluded to avoid treatment-related alterations in imaging characteristics.

### Image acquisition

All CT scans were performed on a PCD-CT scanner (Naeotom Alpha^®^, Siemens Healthineers, Erlangen, Germany). Scans were acquired at 120 kVp in single-source, multi-energy mode (Siemens Healthineers). Images were reconstructed with slice thicknesses of 1 mm and 3 mm. Identical weight-adjusted contrast media protocols were applied: patients < 70 kg received 80 ml iodine contrast at 3.5 ml/s; 70–90 kg, 100 ml at 4 ml/s; and > 90 kg, 120 ml at 4 ml/s (Ultravist^®^ 370, Bayer Vital, Leverkusen, Germany), corresponding to iodine fluxes of 1.3–1.5 gI/s. Each contrast injection was followed by a 50 ml saline flush at 3 ml/s. Bolus tracking for the arterial phase was performed in the proximal abdominal aorta, with a threshold increase of 100 HU. Portal venous phases were acquired with delays of 50 s. From raw data of the venous phase, considered the most reliable phase for the detection of CRLM, VMIs were reconstructed at 40–90 keV in 10 keV increments, along with iodine maps. All reconstructions were based on SPP images (Qr40, ME50) with a slice thickness of 0.8 mm and a slice increment of 0.8 mm.

### Quantitative image analysis

Quantitative analyses were performed by a radiology resident with three years of experience using dedicated workstation software (Syngo.via, VB60, Siemens Healthineers, Erlangen, Germany).

**Fig. 1:**
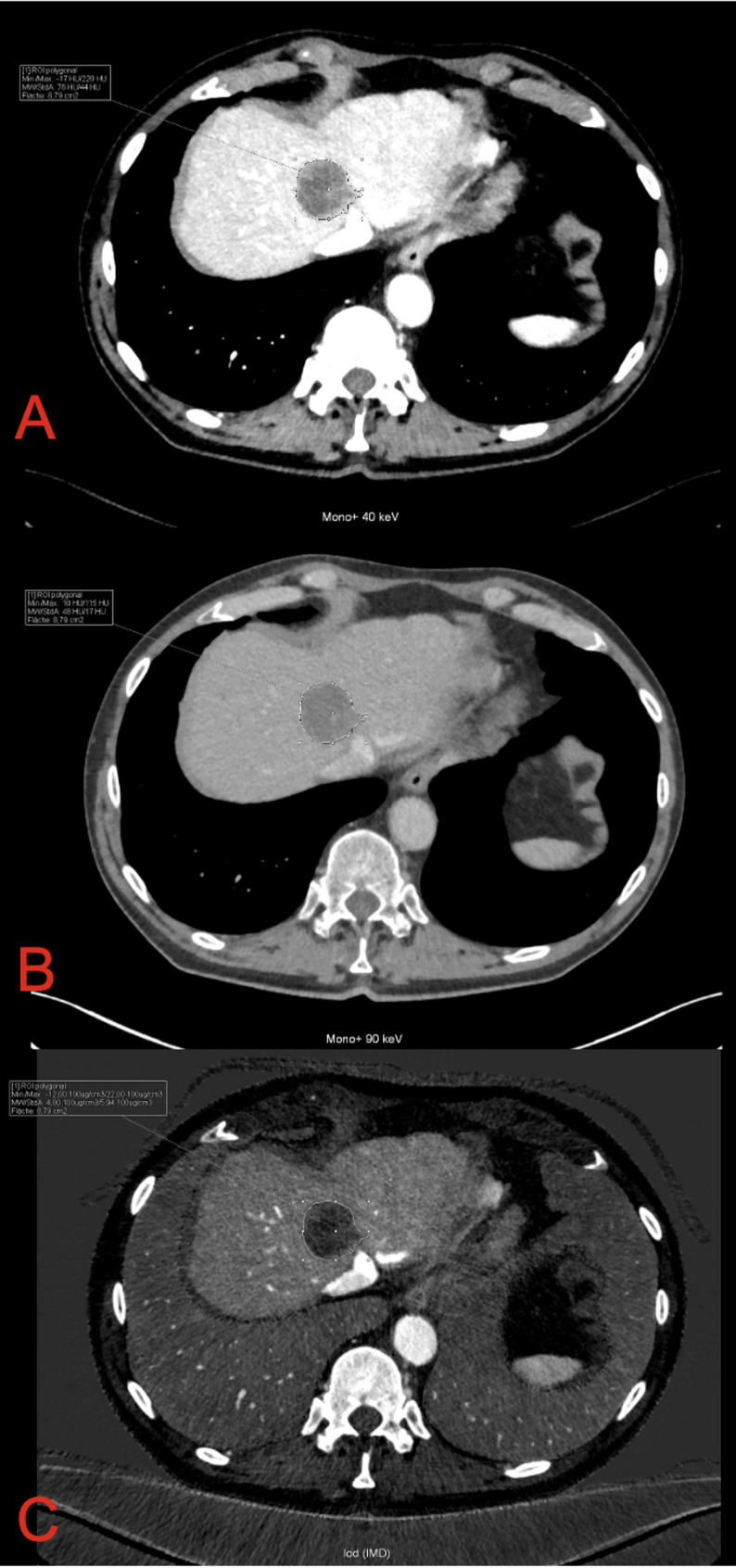
Example of VMI Reconstruction and iodine map of CRLM in Segment VI. A: 40 keV VMI with HU of 76. B: 90 keV VMI with HU of 48. C: Iodine map with IC of 4 100 µg/cm^3^

Density measurements were performed as following: Mean attenuation values (in Hounsfield units (HU)) of metastases and liver parenchyma were obtained for all VMIs reconstructed between 40 and 90 keV. For the iodine map, mean IC (mg/cm^3^) were recorded for metastases and liver parenchyma. Polygonal regions of interest (ROIs) were manually placed on 40 keV images to measure mean attenuation and IC, encompassing the entire metastasis as completely as possible while carefully excluding adjacent vessels (Fig. 1). These ROIs were then propagated across all other reconstructions (including different keV levels and iodine maps) without modification to ensure consistent spatial sampling.

PLR was calculated for each VMI and for the iodine map as the ratio of mean attenuation (in HU) or iodine concentration of the parenchyma to the metastasis

$$\:PLR=\frac{HU\setminus\:Iodine\left(liver\right)}{HU\setminus\:Iodine\:\left(metasasis\right)}$$

CNR was calculated as:

$$\:CNR=\frac{{\upmu\:}\left(\mathrm{l}\mathrm{e}\mathrm{s}\mathrm{i}\mathrm{o}\mathrm{n}\right)-\:{\upmu\:}\left(\mathrm{m}\mathrm{u}\mathrm{s}\mathrm{c}\mathrm{l}\mathrm{e}\right)}{{\upsigma\:}\left(\mathrm{m}\mathrm{u}\mathrm{s}\mathrm{c}\mathrm{l}\mathrm{e}\right)\:}$$

where µ_lesion_ represents the mean attenuation (HU) of the metastasis, µ_muscle_ the mean attenuation of the paravertebral muscle (M. erector spinae), and σ_muscle_ the standard deviation of attenuation in the same muscle ROI as a surrogate for image noise. For the iodine map, lesion and parenchyma iodine concentrations were used analogously.

CNR was used as a quantitative metric, as it reflects lesion-to-background contrast while accounting for image noise and is therefore considered a surrogate marker of lesion conspicuity and detectability [12].

In contrast to prior studies reporting lesion-to-parenchyma ratios [21], we used the inverse formulation (parenchyma-to-lesion ratio), resulting in values greater than 1 for hypodense metastases and thereby facilitating a more intuitive interpretation.

### Subjective image analysis

Three radiologists with 3, 5, and 8 years of experience in abdominal and oncologic imaging, representing a range of expertise from a radiology resident to a board-certified radiologist, independently assessed the visibility of liver metastases, lesion-to-parenchyma contrast, overall image quality, and image noise for the three VMI reconstructions with the highest PLR and CNR (40–60 keV) and iodine maps. Ratings were performed in a blinded fashion using 5-point Likert scales (1 = very poor, 5 = excellent).

### Statistics

All statistical analyses and graphics were performed using R Studio (RStudio Team (2020). RStudio: Integrated Development for R. RStudio, PBC, http://www.rstudio.com, last accessed November 20, 2025) and R 4.0.3 (A Language and Environment for Statistical Computing, R Foundation for Statistical Computing, http://www.R-project.org). Categorical variables were reported as absolute and relative frequencies. Ordinal variables (Likert-scale ratings) were reported as medians and interquartile ranges. Continuous variables were expressed as mean ± standard deviation or median [IQR], as appropriate.

Lesion and liver attenuation across VMIs, the IC, PLR, and CNR were summarized descriptively and visualized. To account for within-patient clustering in lesion-level analyses, linear mixed-effects models were applied with reconstruction level as a fixed effect and random intercepts for patient, with lesions modeled as nested within patients. A paired analysis was performed to test whether the iodine map was superior to the VMI reconstruction with the highest PLR and CNR. The best VMI was defined for CNR and PLR as the keV level showing the highest median value across the dataset. Paired iodine and best-VMI values for each lesion were compared using a one-sided Wilcoxon signed-rank test.

Subjective reader ratings for visibility, contrast, overall image quality, and noise were summarized as median (IQR) for each reconstruction, reported per reader and pooled across readers. Inter-reader agreement was quantified with Krippendorff’s alpha for each criterion and reconstruction. Alpha values were interpreted as: slight (0.0-0.2), fair (0.2–0.4), moderate (0.4–0.6), substantial (0.6–0.8), and near-perfect (0.8-1.0) [22]. A p-value < 0.05 was considered statistically significant.

## Results

### Baseline characteristics

The study included 117 patients with a median age of 65 years (IQR: 57–70). Of these, 41 (35%) were female. CRLM presented synchronously in 75 patients (64%) and metachronously in 42 (36%). At the time of CT, 105 patients (90%) were receiving systemic chemotherapy (Table 1).

**Table 1:** Baseline characteristics of the cohort

Variable	All patients
Age, years, median (IQR)	65 (57–70)
Sex, n (%)	
Female	41 (35%)
Male	76 (65%)
Temporal Presentation, n (%)	
Synchrone	75 (64%)
Metachrone	42 (38%)
Systemic chemotherapy at CT, n (%)	
Yes	105 (90%)
No	12 (10%)
Number of analysed lesions per patient, n (%)	
1 lesion	56 (48%)
2 lesions	12 (10%)
3 lesions	49 (42%)

### Quantitative image analysis

**Fig. 2:**
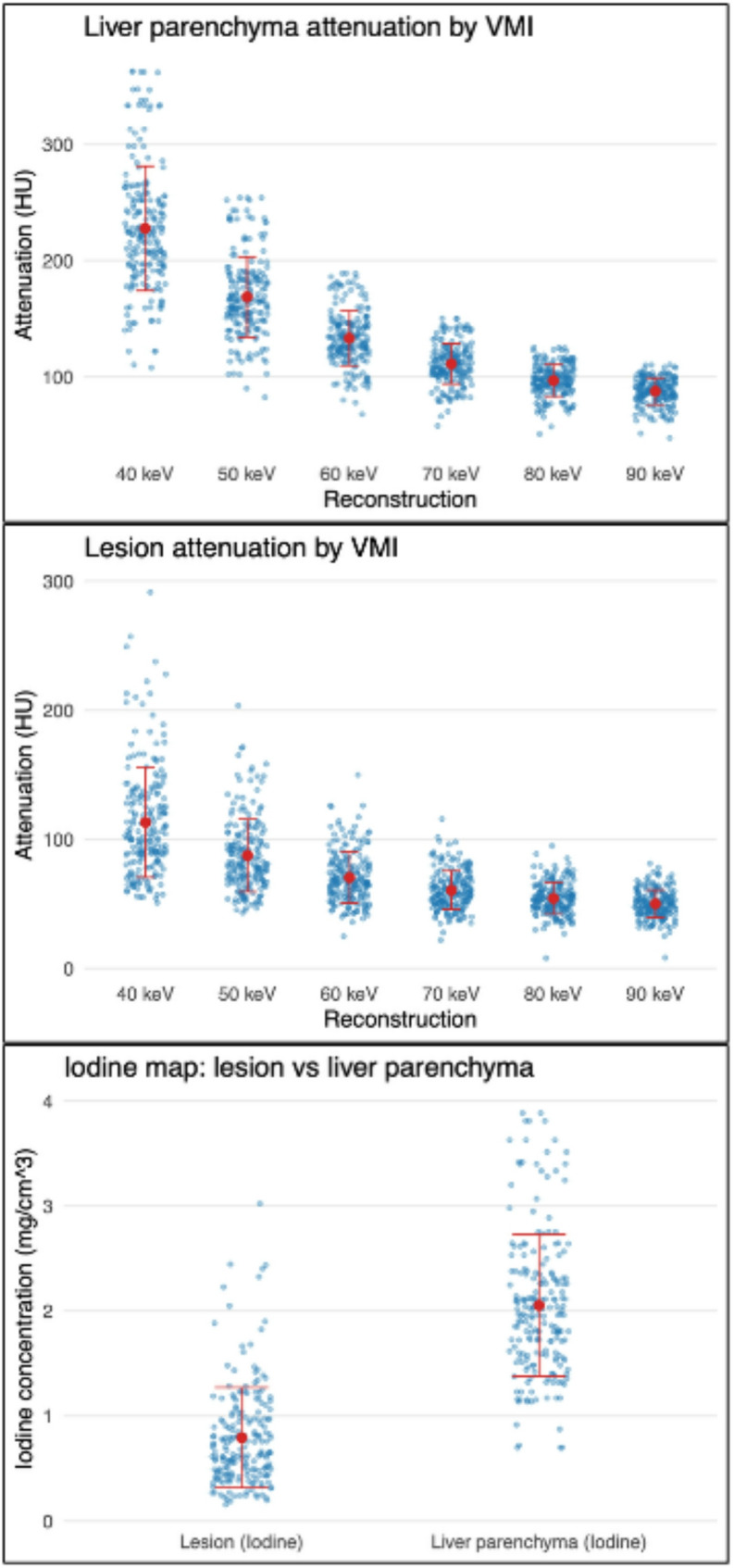
Quantitative HU and IC of liver lesions and liver parenchyma. Scatterplot illustrating individual HU, across the different VMIs, and IC measurements (blue points) derived from PCD-CT. Red markers indicate the group mean, and red vertical error bars represent the standard deviation

Mean lesion attenuation decreased steadily with increasing keV levels, ranging from 113.2 ± 42.6 HU at 40 keV to 54.3 ± 12.4 HU at 80 keV (Fig. 2). Liver attenuation followed a similar trend, with the highest HU values at 40 keV (227.6 ± 53.6 HU) and decreasing toward 96.8 ± 13.6 HU at 80 keV. On the iodine map, mean attenuation values were 0.79 ± 0.48 mg/cm^3^ for lesions and 2.05 ± 0.67 mg/cm^3^ for liver parenchyma.

**Fig. 3:**
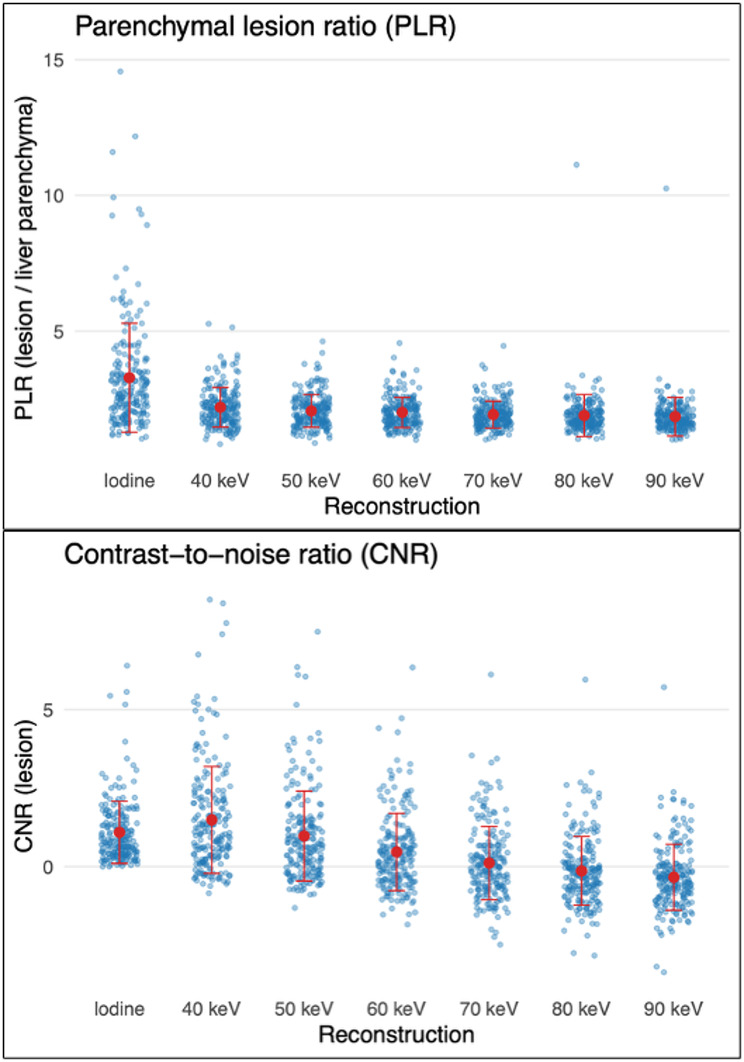
PLR and CNR across iodine maps and virtual monoenergetic reconstructions. Blue points represent individual patient measurements. Red markers indicate the group mean, and red vertical error bars denote the standard deviation within each reconstruction type

The PLR was highest on the iodine map (3.29 ± 2.01), followed by 40 keV (2.19 ± 0.73), with a gradual decrease toward higher energy levels (1.92 ± 0.50 at 70 keV) (Fig. 3).

Linear mixed-effects modeling, accounting for within-patient clustering, demonstrated a significant effect of reconstruction level on PLR (F (6, 1224) = 107.19, *p* < 0.001).

For CNR, the highest lesion-to-background contrast was observed at 40 keV (1.49 ± 1.70), followed by the iodine map (1.09 ± 0.99) and 50 keV (0.97 ± 1.43), with a marked decrease at higher energy levels and substantially lower values at 70–90 keV (Fig. 3).

Linear mixed-effects analysis likewise confirmed a significant effect of reconstruction level on CNR (F (6, 1224) = 423.34, *p* < 0.001).

Using lesion-level paired Wilcoxon superiority tests (one-sided; VMI vs. iodine map), the VMI energy level with the highest median performance across all evaluated VMIs was identified at the dataset level. Based on this criterion, 40 keV emerged as the top-performing VMI. Compared with iodine maps, the 40 keV VMI demonstrated superior CNR, whereas superiority was not met for PLR, which was higher on iodine maps. (Table 2).

**Table 2:** Paired superiority test: Iodine Map vs. best VMI

Endpoint	Best VMI (keV)	Paired *n*	Median Δ (best - iod)	Wilcoxon *p*
PLR	40	227	-0.654	1
CNR	40	227	+ 0.289	< 0.001

### Subjective image analysis

**Fig. 4:**
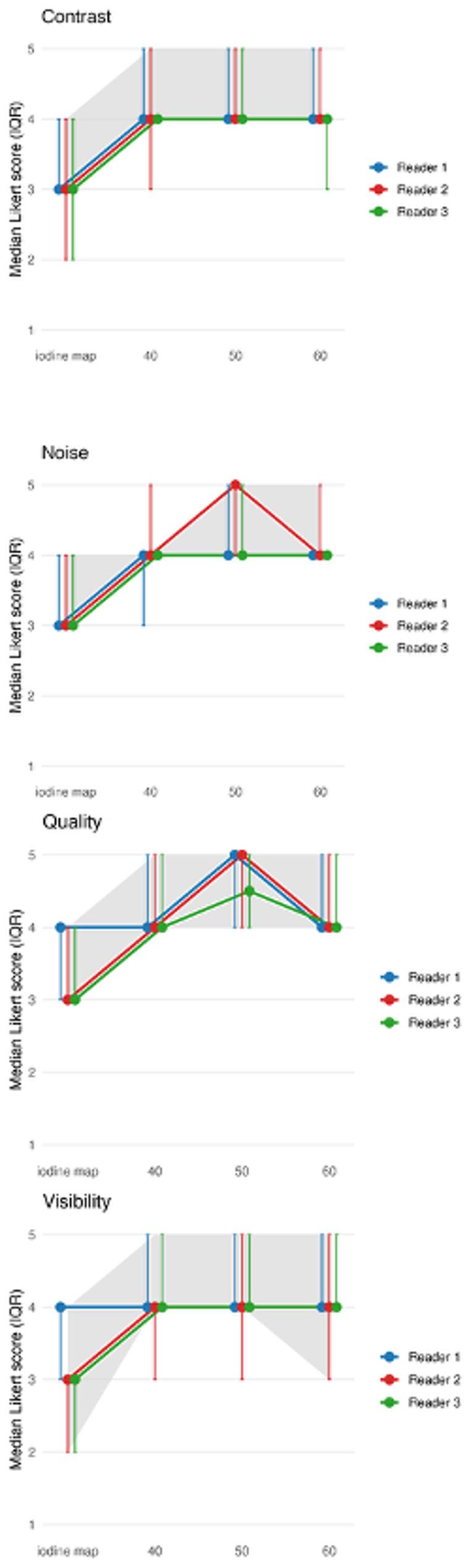
Subjective image quality trends (visibility, contrast, quality, and noise) across monoenergetic levels (40–60 keV). Shaded area = pooled interquartile range (Q1-Q3). Lines may overlap where identical median Likert scores were assigned

Across all three readers, median ratings favored lower-keV VMIs. 50 keV achieved the highest median for overall image quality, while 40–60 keV achieved similarly high medians for visibility and contrast (Fig. 4). The iodine map scored consistently lower across all criteria. The pooled IQR across readers was generally narrow, with the tightest clustering for noise at 40 keV ([4–4]) (Table 3), indicating high consistency of ratings in that setting.

**Table 3:** Subjective rating (Median (IQR)

Quality criterion	Iodine map	VMI 40 keV	VMI 50 keV	VMI 60 keV
Visibility	3 (2–4)	4 (4–5)	4 (4–5)	4 (3–5)
Contrast	3 (3–4)	4 (4–5)	4 (4–5)	4 (4–5)
Overall image quality	3 (3–4)	4 (4–5)	5 (4–5)	4 (4–5)
Noise	3 (3–4)	4 (4–4)	4 (4–5)	4 (4–5)

**Fig. 5:**
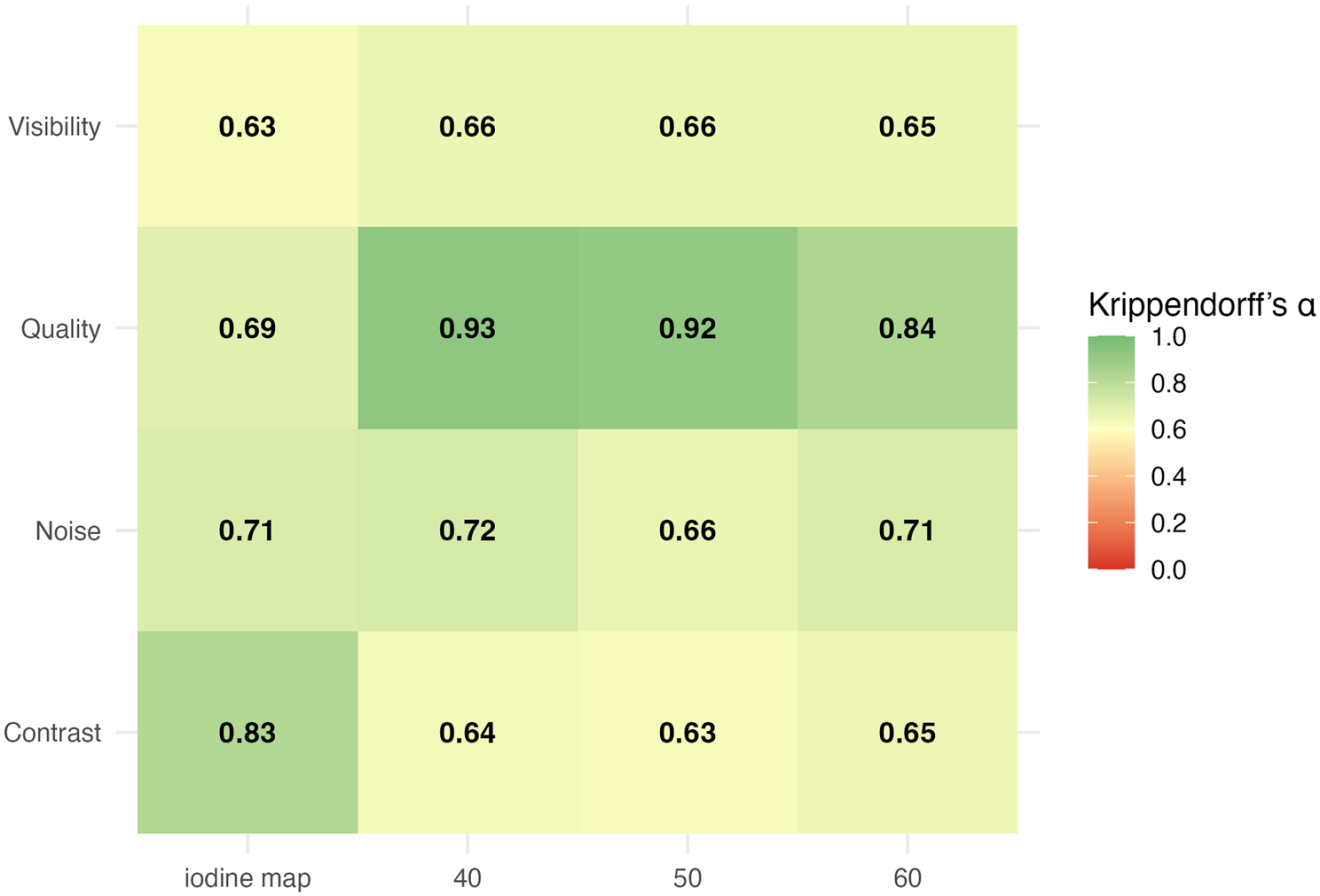
Krippendorf’s alpha (Inter-reader agreement)

Inter-reader agreement was high across all criteria, with a minimum of substantial agreement. The highest concordance was observed for overall image quality at 40 and 50 keV. Visibility, noise, and contrast showed substantial to near-perfect agreement, ranging from α = 0.63 to α = 0.83 across reconstructions (Fig. 5).

## Discussion

This study demonstrates that PCD-CT provides complementary advantages in the evaluation of CRLM when combining low-keV VMIs and iodine maps, in both objective and subjective analysis.

In objective analyses, low-keV VMIs significantly increased the CNR. This finding aligns with previous studies, such as that by Bette et al., who demonstrated improved CNR of liver metastases at low keV [21]. Despite using a different CNR calculation method (standard deviation of subcutaneous fat) and a heterogeneous cohort including various types of liver metastases (13 out of 50 were CRLM), they also identified 40 keV as the optimal energy level for CNR [21].

Likewise, Bette et al. showed that among VMIs, 40 keV achieved the highest attenuation ratio between tumour and liver parenchyma, with a reported median tumour-to-liver ratio of 0.37 (0.27–0.53). When expressed according to our inverse definition (parenchyma-to-lesion ratio, PLR), this corresponds to approximately 2.7 (IQR 1.9–3.7), which is in the same range as the PLR observed in our CRLM-specific cohort at 40 keV (2.19 ± 0.73), thereby extending these findings to a larger and entity-focused population.

However, our study demonstrated that the iodine map achieved a superior PLR compared with the 40 keV VMIs. Sartoretti et al. demonstrated that iodine quantification in the liver, including liver metastases, is accurate and robust across varying doses and iodine concentrations, supporting the reliability of iodine-based metrics [17]. Paired superiority testing confirmed this complementarity: at 40 keV, VMIs outperformed iodine maps in terms of CNR, while the iodine map was superior for PLR.

In contrast to previous studies by Bette et al. and Bae et al., which exclusively analysed virtual monoenergetic images, our study additionally incorporated iodine maps into the evaluation [15, 21]. This combined analysis enabled a direct comparison of spectral and iodine-based reconstructions within the same patient cohort, highlighting their complementary diagnostic capabilities in photon-counting CT. Clinically, these results support a dual-track reading approach in PCD-CT: using low-keV VMIs (40–50 keV) to improve CNR and subjective image quality and iodine maps for visibility and characterization based on PLR. Future studies should further investigate whether this combination, together with optimized reconstruction techniques, can enhance diagnostic confidence and whether PCD-CT can approach the sensitivity of MRI, which currently remains the most sensitive modality for detecting and characterizing CRLM.

Subjective ratings closely mirrored the quantitative results. Across readers, overall image quality peaked around 50 keV, and visibility and contrast were rated highest between 40 and 60 keV. Despite its high PLR, the iodine map achieved lower subjective scores, which could in part be explained by reader familiarity. Radiologists are generally more accustomed evaluating conventional CT images, whereas iodine maps present a different visual texture. This unfamiliar appearance may have influenced subjective perception, leading to slightly lower visual or diagnostic ratings despite favourable quantitative parameters. Cognitive biases are a well-recognized factor influencing image interpretation and reader perception in radiology. As discussed by Yoon et al. and Zhang et al., diagnostic decision-making can be affected by prior experience, expectations, and perceptual familiarity with certain image appearances or modalities [23, 24]. Such biases may lead radiologists to rate image types they are accustomed to, such as conventional CT or VMIs, more favourably than less familiar representations such as iodine maps. Similar familiarity-driven effects have also been demonstrated outside of radiology, where individuals tend to evaluate familiar stimuli or ideas more positively than novel ones [25, 26]. This suggests that the lower subjective preference for iodine maps observed in our study may, at least in part, reflect a general familiarity-related perceptual tendency rather than a true limitation of the imaging technique itself. Similar discrepancies between objective and subjective assessments have been observed in prior studies such as iodine maps in CT pulmonary angiography [27].

The combined use of low-keV VMIs and iodine maps may offer clinically relevant benefits beyond routine lesion detection. Improved conspicuity of small metastases using low-keV VMIs could be particularly valuable for preoperative planning, where accurate identification of lesion number and distribution directly influences surgical strategy [5].

In addition, the quantitative nature of iodine maps may enable objective assessment of lesion vascularity, providing a basis for longitudinal treatment monitoring and therapy response evaluation. Beyond colorectal liver metastases, Müller et al. demonstrated that PCD-CT shows higher concordance with MRI for LI-RADS classification of liver lesions than conventional CT, suggesting that advanced spectral information from PCD-CT may narrow the diagnostic gap between CT and MRI in hepatic oncology [28].

Several limitations must be acknowledged. This was a retrospective, single-center analysis conducted on one PCD-CT platform, which may limit generalizability. Restricting analysis to a maximum of three lesions per patient may not fully eliminate intra-patient clustering effects [29] and may introduce a bias toward larger and more conspicuous lesions, whereas the detection of small lesions remains a clinically relevant challenge. Furthermore, as the majority of patients were undergoing systemic chemotherapy at the time of imaging, the generalizability of the findings to untreated patients may be limited. Only one examination per patient was evaluated, limiting assessment of PLR as a longitudinal biomarker. In future projects, we plan to perform longitudinal analyses of PLR in serial photon-counting CT examinations to explore its potential as a quantitative marker for treatment response and disease monitoring. Additionally, subjective results could evolve as radiologists gain experience with iodine maps, potentially narrowing the current gap between quantitative and qualitative assessments.

## Conclusion

PCD-CT shows complementary strengths when combining low-keV VMIs and iodine maps for the evaluation of colorectal liver metastases. Low-keV VMIs enhance CNR and subjective image quality, whereas iodine maps provide superior PLR. A protocol using 50 keV for general review, 40 keV for improved CNR and iodine maps for visibility appears both effective and clinically practical.

## Data Availability

The datasets generated and analysed during the current study are not publicly available due to them containing information that could compromise patient privacy. However, data are available from the corresponding author on reasonable request.

## Abbreviations

CNR: Contrats-to-noise-ratio
CRC: Colorectal cancer
CRLM: Colorectal liver metastases
CT: Computed tomography
EID-CT: Energy-integrating detector CT
HU: Hounsfield units
IC: Iodine concentration
IQR: Interquatile range
MRI: Magnetic resonance imaging
PCD-CT: Photon-counting setector CT
PLR: Parenchyma-to-lesion ratio
ROI: Region of interest
VMI: Virtual monoenergetic imaging
